# Challenging behaviour and its risk factors in children and young people in a special school setting: A four wave longitudinal study

**DOI:** 10.1111/jar.13066

**Published:** 2022-12-23

**Authors:** Gemma Nicholls, Tom Bailey, Corinna F. Grindle, Richard P. Hastings

**Affiliations:** ^1^ Calthorpe Academy Birmingham UK; ^2^ Centre for Educational Appraisal, Research & Development (CEDAR) University of Warwick (UK) Coventry UK

**Keywords:** challenging behaviour, intellectual disabilities, longitudinal design, special schools

## Abstract

**Background:**

Longitudinal research is needed to strengthen evidence for risk factors for challenging behaviour in children with intellectual disabilities and to understand patterns of change over time.

**Methods:**

Data on challenging behaviour were collected for 225 students in one school over four annual time points and a range of potential risk correlates. Data were analysed using Generalised Estimating Equations.

**Results:**

Prevalence of challenging behaviour, aggression and self‐injury did not vary significantly over time. Stereotyped behaviours increased over the 4‐year period. Challenging behaviour was associated with lower levels of adaptive skills and autism. Stereotyped behaviour increased with age. Self‐injurious behaviour was less likely to be shown in children with profound intellectual disabilities over time.

**Conclusions:**

These findings are consistent with previous research in terms of potential risk factors identified. Implications for schools include proactive interventions for children with intellectual disabilities at high risk; especially those with autism and poorer adaptive skills.

## 1 INTRODUCTION

Children with intellectual disabilities have an increased risk of developing behaviours that may be considered by others as challenging (Hastings et al., 2013). Total population studies have indicated that prevalence of challenging behaviour in the intellectual disabilities population ranges from 10% to 18% (Emerson, Kiernan, Alborz, et al., 2001; Holden & Gitlesen, 2006; Lundqvist, 2013). Studies specifically assessing children with intellectual disabilities and challenging behaviour in special school populations have shown that prevalence can range between a quarter of the population (22.2%, Kiernan & Kiernan, 1994; 25%, Male & Raynor, 2009) to up to 82% of the population (Murphy et al., 2009). Recently, Nicholls et al. (2020) used a rigorous assessment method derived from a standard challenging behaviour screening tool and found that 53% of students with an intellectual disability in one large special school had challenging behaviour. Given that the effects of challenging behaviour are typically negative for the child and the people supporting them (Emerson, Kiernan, Emerson, et al., 2001; Holden & Gitlesen, 2006; Sugai et al., 2000), and children with challenging behaviour are likely to also display challenging behaviour in adulthood (Murphy et al., 2005), it is important to understand the factors that contribute to challenging behaviour and which children are at a higher risk.

Cross‐sectional studies on children with intellectual disabilities and challenging behaviour have identified a number of potential risk factors as they have been found to be associated with challenging behaviour outcomes. These factors have included: the presence of autism (Mazurek et al., 2013; Nicholls et al., 2020), lower levels of adaptive skills (Nicholls et al., 2020), lower levels of communication skills (Kiernan & Kiernan, 1994), severity of intellectual disability (Murphy et al., 2009), and deprivation experienced by the child's family (Nicholls et al., 2020). However, to generate strong evidence for the risk factors for challenging behaviour, longitudinal study designs are needed. There are though relatively few longitudinal studies of challenging behaviour in children with intellectual disabilities.

A follow up study of children with severe intellectual disabilities was conducted by Chadwick et al. (2005). They recruited 82 children aged 4–11 years from four schools for children with severe intellectual disabilities and two schools for children with mild intellectual disabilities in three inner London boroughs, and repeated data collection 5 years later. Chadwick et al. (2005) conducted parental interviews using the Behaviour Problems section of the Disability Assessment Schedule (Wing, 1989) to collect information on the presence and severity of behaviour problems. An overall score for challenging behaviour was determined by combining the ratings across nine individual behaviours. Chadwick et al. also administered parent and teacher questionnaires using the Aberrant Behaviour Checklist (ABC, Marshburn & Aman, 1992). The persistence rate of behaviour problems including self‐injury, aggression and destruction was 89.7% but correlates of challenging behaviour over time were not examined.

Davies and Oliver (2016) reported repeated data over 15–18 months in a sample of 417 children between the ages of two and 12 with severe intellectual disabilities recruited from 14 special schools. They administered the Self‐injury, Aggression and Destruction Screening Questionnaire (SAD‐SQ, Davies & Oliver, 2016). This uses a Likert scale for severity and frequency looking specifically at self‐injury, aggression and destruction as well as severity of intellectual disability, repetitive and restricted behaviours and impulsive behaviours. For most children, challenging behaviour was persistent: Self‐injury (58%), aggression (69%) or destruction (57%) was either still present or continued to not be demonstrated at the second time point. Severity of intellectual disability, age and gender were not significantly associated with challenging behaviour cross‐sectional at the first data collection point. However, repetitive and restricted behaviours and interests predicted the cumulative presence of self‐injurious behaviour and aggressive destructive behaviours 15–18 months later. Overactivity and impulsivity predicted the presence of aggressive destructive behaviour over time.

Focussing on young children who were at risk for developmental delay, Rojahn et al. (2016) used a longitudinal design at three time points in a 1‐year period. Data came from a longitudinal research project in Peru where parents had expressed concerns about their child's developmental delays and related problems with behaviour. Children were included if they had risk factors for genetic intellectual disabilities, developmental delays, family history of disabilities or behaviour disorders. Data were collected for 160 children ranging from 4 to 44 months of age. Seventy children were considered to have autism and 90 had other developmental delays associated with conditions such as Down Syndrome. Rojahn et al. examined stereotypic behaviour and self‐injurious behaviour through parents completing the Behaviour Problems Inventory (BPI 01, Rojahn et al., 2001) and the Bayley Cognitive Scale (Bayley, 2006), completed through observation. Children were more likely to show self‐injurious behaviour at later time points if they had demonstrated more frequent stereotypic behaviour in the earlier assessments.

In the existing research, there are few longitudinal studies focussing on children in a special school context; and the studies that do exist typically focus on two time points only (with Rojahn et al., 2016 a notable exception but over a short time scale). Existing research studies also have little focus on examining the associations with risk factors over time, and there is a lack of consistency in the use of challenging behaviour measures and research methods. Multiple time point longitudinal designs are needed to understand more about how challenging behaviour fluctuates over longer periods, and what factors are associated with changes over time in challenging behaviour. Although there is existing multiple time point research in children with intellectual disabilities, this does not focus specifically on challenging behaviour but rather on behavioural and emotional problems more broadly. For example, Bailey et al. (2019) plotted the trajectories of behavioural and emotional problems in children with intellectual disabilities over five time points from age 3 to 11 years in a UK cohort study. They concluded that for children with intellectual disabilities, prosocial behaviours improved between age 3 and 11 years, although there was a slowing of the trajectory between ages five and seven. They also concluded there was no change over time in internalising behaviour problems between 3 and 11 years. There was no significant covariance between maternal mental health and behaviour problems. However, there was a positive covariation between maternal distress and internalising and externalising behaviours. When maternal distress was higher, child internalising and externalising behaviour problems were higher.

The main aim of the current study was to examine challenging behaviour over four annual time points and to consider risk markers for challenging behaviour over time in a special school population. Due to current research lacking consistency in the use of challenging behaviour measures, this study adopted definitions of challenging behaviour from previous research (Nicholls et al., 2020). Given the results of existing cross‐sectional and longitudinal research, we examined a range of potentially important correlates or risk markers for challenging behaviour over time, specifically: child age, adaptive skills, presence of autism, presence of profound intellectual disability, whether they are a child in care, English as language spoken at home, and neighbourhood deprivation associated with the family's residence.

## 2 METHOD

### 2.1 Participants

Participants were 225 students (164 male and 61 female) who attended one special school between July 2016 and July 2019. This was an all through special school where students' ages ranged from 4 to 19 years old. The school accepts students who have an Education, Health Care Plan in place with a range of Special Educational Needs ranging from severe learning disabilities (i.e., severe intellectual disability) to profound intellectual disabilities plus additional needs such as autism. Using school records, participants were recorded as having autism or not, and Profound intellectual disability or not. All participants with autism also had a Severe Learning Disability (i.e., severe intellectual disability). Severe intellectual disability is characterised by having little or no speech, difficulty in learning new skills, needing support with daily activities, difficulty with social skills and likely needing life‐long support. Autism was the most prevalent primary need for the children as recorded by the school (46.7% of the sample). A primary need of profound intellectual disability was recorded for 18.7% of students and severe intellectual disability only was recorded for 34.6%. English was the language spoken at home for only 41.3% of participants. Characteristics of the participants is summarised in Table 1. The Index of Multiple Deprivation (Ministry of Housing, Communities and Local Government, 2019) was used to estimate the level of deprivation experienced by participants based on postal code information for the neighbourhood in which they lived. The IMD is as an official measure of relative deprivation for small areas combining 37 separate indictors grouped into seven domains (income, employment, education, health deprivation/disability, crime, barriers to housing/services, living environment). Combining this information gives an overall relative measure of deprivation. A large majority (82.7%) of participants lived in areas that were in the 20% most deprived neighbourhoods in England.

**TABLE 1:** Characteristics of students

Characteristic	Summary statistic, *N* (%)
Gender	
Male	164 (72.9%)
Female	61 (27.1%)
Language spoken at home	
English	93 (41.3%)
Other than English	132 (58.7%)
Primary need	
ASC	105 (46.7%)
Severe ID	78 (34.6%)
Profound ID	42 (18.7%)
Child in care	
In care	13 (5.8%)
Not in care	212 (94.2%)

### 2.2 Measures

#### 2.2.1 The behaviour problems inventory‐short form‐school version (BPI‐S‐school)

Rojahn et al. (2012) developed the Behaviour Problems Inventory‐Short Form (BPI‐S) to measure challenging behaviour in individuals with intellectual disabilities over the previous 6 months. This is a shorter version of the Behaviour Problems Inventory‐01 (BPI‐01) (Rojahn et al., 2001). The BPI‐S‐Schools (Nicholls et al., 2020) has 32 questions divided into three categories: Self‐injurious Behaviour (10 items), Aggressive Destructive Behaviour (10 items), and Stereotyped Behaviour (12 items). The BPI‐S‐Schools version includes some amendments to the BPI‐S relevant to students with intellectual disabilities in school settings. Two additional items were added to the self‐injurious behaviour subscale (self‐pinching and skin picking), and bullying was amended to ‘grabbing items from others (e.g., toys or food)’. Nicholls et al. (2020) reported good internal consistency of the BPI‐S‐Schools. Cronbach's *α* for the total BPI‐S‐Schools adapted version total frequency score was .93 and .90 for severity. The Cronbach's *α* coefficients for the self‐injurious behaviour subscale were .77 for frequency and .83 for severity. The *α* coefficients for the aggressive/destructive behaviour subscale for frequency were .89 and .89 for severity. The *α* coefficient for the stereotyped behaviour subscale, for frequency scores only, was .91.

For both self‐injurious behaviour and aggressive/destructive behaviour subscales the BPI‐S‐Schools has two Likert rating scales: a 5‐point frequency scale (0 = never; 1 = monthly; 2 = weekly; 3 = daily; 4 = hourly) and a 3‐point severity scale (1 = mild; 2 = moderate; 3 = severe) with clearly described anchor points. The stereotyped behaviour subscale is measured only on frequency, using an 8‐point frequency scale (0 = never; 1 = fewer than once a month; 2 = about once per month; 3 = about once per week; 4 = about once per day; 5 = about once per hour; 6 = more than once per hour; 7 = once per minute or more).

#### 2.2.2 Great outcomes for kids impacted by severe developmental disabilities (GO4KIDDS) brief adaptive scale

The *GO4KIDDS* is a brief measure of adaptive behaviour for children and adolescents with severe intellectual disabilities (Perry et al., 2015). The measure has eight items: support needs, understanding of spoken language, use of spoken language to communicate, engagement in social interactions with familiar adults, engagement in social interaction with other children, and the child's skills in eating, toileting, and dressing. Each item is rated on a 5‐point scale, with one requiring support for almost all aspects and five demonstrating greater independence. For example, for the support needs item (‘What level of help or support is needed for this child (e.g., toileting, dressing, eating?)’) the rating is as follows: 1 = requires support for almost all aspects of life; 2 = requires support for most, but not all, aspects of life; 3 = requires support for some aspects of life; 4 = requires support for only a few aspects of life; 5 = does not require support. These 1–5 scores across items are then summed to obtain an overall Adaptive Behaviour score. Pan et al. (2019) reported an internal consistency for the GO4KIDDS total score as .93 (Cronbach's *α*) for students in a special school setting and that the structural validity of the measure was good as the scores were an adequate reflection of the adaptive skills of participants. This, along with findings from Perry et al. (2015) (Cronbach's *α* was .87) demonstrate that GO4KIDDS can reliably measure adaptive skills.

### 2.3 Working definition of challenging behaviour

The definition of challenging behaviour used in the research replicated the definitions used in Nicholls et al. (2020, p. 47):

- ‘Self‐injurious behaviour: Any self‐injurious behaviour is “challenging” if either it is rated as severe, or is rated as moderate and occurs at least weekly, or is rated as mild and occurs at least daily. Any other occurrence of self‐injurious behaviour is not rated as challenging.
- Aggressive/destructive behaviour: Any aggressive destructive behaviour is “challenging” if either it is rated as severe, or is rated as moderate and occurs at least weekly, or is rated as mild and occurs at least daily. Any other occurrence of behaviour is not rated as challenging.
- Stereotyped behaviour: Any stereotyped behaviour is “challenging” if it occurs more than once per hour. Any other occurrence of behaviour is not rated as challenging.
- Challenging behaviour: Overall challenging behaviour is defined by the presence of a least one behaviour defined as “challenging” in the categories a–c.’

### 2.4 Procedure

Standard data were collected by the school for all students on entry, for monitoring and information purposes. Date of birth, gender, postcode, ethnicity, language spoken at home, and primary educational need for each student was extracted from these school records in July 2016. Data were collected for all students enrolled in the school at the end of each academic year. Students who were at the school for all four time points (*N* = 225) were included in this study.

The initial sample at time point one was 321 students. Over the four time points students who finished their placement at year 14 (age 19) left the school and therefore were no longer part of the ongoing data collection. The resulting sample was therefore 225 students who were at the school for all four‐time points. There were no other missing data as teachers were asked to complete any missing items each year.

Training was delivered to all class teachers where they were provided with an explanation on how to complete the measures (BPI‐S‐Schools and GO4KIDDS) and the purpose of the data collection. Teachers completed the measures for each student in their class at the end of each academic year between July 2016 and July 2019. They knew each student well as they had worked with them typically for 6 h/day throughout the year. New teachers to the school received the same initial training immediately prior to each data collection point. Because students changed classes and teachers annually, different teachers completed the measures each year for the same student.

### 2.5 Approach to data reduction and analysis

Data were analysed using SPSS Version 25 (IBM Corp, Version 25.0. Armonk, NY). Generalised estimating equations (GEE) examined the association between the presence of challenging behaviour and age, adaptive skills, presence of autism, and presence of profound intellectual disability. The data were repeated measures and therefore non‐independent; this was accounted for with GEEs which estimate population average effects. For the models reported here, all the outcomes were binary.

Step one used time variant predictors (i.e., age and adaptive skills that varied over time) with the addition of time invariant (i.e., fixed/single time point) predictors in step two. The first conditional growth model looked at age and adaptive skills over all time points as predictor variables as adaptive skills were measured at each time point. The second conditional growth model looked at age, adaptive skills, gender, presence of autism, presence of profound intellectual disability, whether they were a child in care, English as language spoken at home, and neighbourhood deprivation as predictor variables. All predictors other than age and adaptive skills were as reported at the first data collection point.

Variables from the demographic information were collated into categories for analysis. Binary variables were created: autism versus no autism, Profound intellectual disability versus no Profound intellectual disability, and ‘English’ versus ‘other than English’ for language spoken at home. The total score for adaptive skills was used from the GO4KIDDS measure. Date of birth was converted to age at each time point. To determine level of deprivation, postcodes were converted to Ministry of Housing, Communities and Local Government (2019) decile then a dichotomous variable was created indicating whether students were living in one of the 20% most deprived neighbourhoods in England.

The four outcomes were recoded into binary variables from BPI‐S‐Schools data in accordance with the definitions of challenging behaviour, using SPSS syntax. For example, self‐injurious behaviour was coded as present (score one) if the student's BPI scores met criteria for the definition of self‐injurious behaviour, and the student was coded as zero for self‐injurious behaviour otherwise. Prevalence at each time point was described as a percentage with 95% confidence intervals on the basis of the proportion of the sample meeting the definitions described.

## 3 RESULTS

### 3.1 Prevalence of challenging behaviour

When the definitions derived from the BPI‐S‐Schools were applied 58.2% (95% CI [51.6, 64.9]) of participants met criteria for challenging behaviour overall (*n* = 131) at time point one. There was some variation over time points and at time point four prevalence of challenging behaviour had increased to 64% (95% CI [57.8, 70.2]). However, there was no clear upwards or downwards trend and the confidence intervals at all time points overlapped suggesting no clear change in prevalence over time. Self‐injurious behaviour was most prevalent at time point one: 41.3% (95% CI [34.7, 48]). This decreased over time to 37.3% (95% CI [31.1, 43.6]) at time point two, then 37.3% (95% CI [31.1, 43.6]) by time point four. There was no clear upwards or downwards trend and the confidence intervals at all time points overlapped suggesting no clear change in prevalence over time. Aggressive/destructive behaviour was displayed by 33.3% (95% CI [27.1, 39.6]) of children at time point one, before increasing to 40.4% at time point four (95% CI [33.8, 47.1]). Again, there was no clear upwards or downwards trend and the confidence intervals at all time points overlapped suggesting no clear change in prevalence over time. Stereotyped behaviour was displayed by 28.4% (95% CI [22.7, 34.7]) of children at time point one with a gradual increase over all time points to 42.2% (95% CI [36.0, 48.9]) by time point four. As there were no overlap between confidence intervals for prevalence at times one and four, there was evidence of a significant increase over time in the prevalence of stereotyped behaviour. The prevalence of each subtype of challenging behaviour at each time point is summarised in Table 2.

**TABLE 2:** Prevalence of challenging behaviour of each subtype at each time point

Category	Prevalence (%) [95% CI]			
	Time 1	Time 2	Time 3	Time 4
Self‐Injurious behaviour	41.3 [34.7, 48.0]	37.3 [31.1, 43.6]	37.8 [31.6, 44.0]	37.3 [31.1, 43.6]
Aggressive‐destructive behaviour	33.3 [27.1, 39.6]	32.4 [26.7, 38.7]	39.1 [32.9, 45.3]	40.4 [33.8, 47.1]
Stereotyped behaviour	28.4 [22.7, 34.7]	34.7 [28.9, 40.9]	36.9 [30.7, 43.1]	42.2 [36.0, 48.9]
Overall challenging behaviour	58.2 [51.6, 64.9]	57.8 [51.6, 64.4]	61.3 [55.1, 67.6]	64.0 [57.8, 70.2]

### 3.2 Predictors of change over time in challenging behaviour

The results of the generalised estimating equations analysis are summarised in Table 3. Data on age and adaptive skills were available at all four time points. All other variables were measured at time one only.

**TABLE 3:** Generalised estimating equations for challenging behaviour and risk correlates

	Challenging behaviour				Self‐injurious behaviour				Aggressive destructive behaviour				Stereotyped behaviour			
	*B* (95% CI)	SE	Chi‐square	*p*	*B* (95% CI)	SE	Chi‐square	*p*	*B* (95% CI)	SE	Chi‐square	*p*	*B* (95% CI)	SE	Chi‐square	*p*
Model 1: Age																
Age	.057 (.004, .110)	.0270	4.383	.036	.016 (−.036, .068)	.0263	.371	.542	.049 (.000, .099)	0.253	3.811	.51	.105 (.056, .154)	.0249	17.869	<.001
Model 2: Age and adaptive skills																
Age	.081 (.025, .137)	.0286	8.075	.004	.047 (−.007, .100)	.0273	2.922	.087	.060 (.009, .110)	.0258	5.417	.020	.118 (.068, .167)	.0253	21.569	<.001
Adaptive skills	−.050 (−.074, −.027)	.0121	17.350	<.001	−.068 (−.091, −.045)	.0115	34.649	<.001	−.025 (−.047, −.002)	.0115	4.601	.032	−.029 (−.051, −.007)	.0112	6.743	.009
Model 3: All predictors																
Age	.077 (.019, .136)	.0300	6.655	.010	.035 (−.019, .089)	.0276	1.613	.204	.054 (.002, .105|)	.0263	4.151	.042	.135 (.082, .187)	.0268	25.376	<.001
Adaptive skills	−.100 (−.128, −.073)	.0143	49.643	<.001	−.099 (−.126, −.072)	.0138	52.115	<.001	−.044 (−.070, −.018)	.0133	11.114	.001	−.070 (−.097, −.044)	.0134	27.538	<.001
Gender	.105 (−.396, .606)	.2558	.168	.682	.153 (−.395, .701)	.2795	.298	.585	.177 (−.311, .665)	.2491	.506	.477	−.332 (−.783, .119)	.2303	2.077	.150
Presence of ASC	1.126 (.670, 1.582)	.2326	23.421	<.001	.864 (.385, 1.354)	.2498	11.976	.001	.738 (.274, 1.201)	.2366	9.724	.002	1.251 (.808, 1.694)	.2261	30.623	<.001
Presence of PMLD	−1.275 (−2.004, −.545)	.3722	11.732	.001	−.855 (−1.626, −.084)	.3935	4.722	.030	−.489 (−1.224, .246)	.3750	1.702	.192	−.723 (−1.481, .034)	.3865	3.501	.061
Child in care	−1.123 (−2.482, .236)	.6934	2.623	.105	−.943 (−1.940, .053)	.5084	3.442	.064	−.567 (−1.463, .330)	.4572	1.535	.215	−.135 (−.720, .450)	.2984	.205	.651
English as home language	.250 (−.217, .717)	.2383	1.099	.295	.103 (−.348, .554)	.2301	.202	.653	.181 (−.244, .606)	.2167	.697	.404	.189 (−.204, .582)	.2006	.885	.347
Level of deprivation (IMD)	−.096 (−.664, − .473)	.2902	.108	.742	−.284 (−.839, .271)	.2831	1.008	.315	.029 (−.573, .631)	.3071	.009	.925	−.046 (−.523, .431)	.2434	.035	.851

There was no statistically significant effect of age on self‐injurious behaviour over time (*B* = .016 [−.036, .068], *p =* .542). However, at time points when adaptive skills were higher, self‐injurious behaviour was less likely to occur (*B* = −.068 [−.091, −.045] *p =* <.001). The presence of self‐injurious behaviour over time was more likely when autism was present (*B* = .864 [.385, 1.354], *p* = .001). Children with a profound intellectual disability were less likely to show self‐injurious behaviour over time (*B* = −.855 [−1.626, −.084], *p =* .030).

There was also no statistically significant relationship between age and aggressive/destructive behaviour over time (*B* = .049 [.000, .099], *p =* .51). The presence of aggressive/destructive behaviour over time was also more likely when autism was present (*B* = .738 [.274, 1.201], *p =* .002). At time points where adaptive skills were higher, again aggressive/destructive behaviour was less likely to occur (*B* = −.044 [−.070, .018], *p* = .001). There was no statistically significant relationship between aggressive/destructive behaviour and the presence of profound intellectual disability (*B* = −.489 [−1.224, .246] *p =* .192).

The presence of stereotyped behaviour increased with age (*B* = .105 [.056, .154], *p =* <.001). At time points where adaptive skills were higher, stereotyped behaviour was again less likely to occur (*B* = −.029 [−.051, −.007], *p =* .009). The presence of stereotyped behaviour over time was again more likely when autism was present (*B* = 1.251 [.808, 1.694], *p =* <.001). There was no statistically significant relationship between the presence of profound intellectual disability and stereotyped behaviour over time (*B* = −.723 [−1.481, .034], *p =* .061).

There was also a statistically significant relationship between age and challenging behaviour over time (*B* = .057 [.004, .110] *p =* .036) with the presence of challenging behaviour increasing with age. At time points when adaptive skills were higher, challenging behaviour was again less likely to occur (*B* = −.050 [−.074, −.027] *p =* <.001). Challenging behaviour was also more likely when autism was present (*B* = 1.126 [.670, 1.582], *p =* <.001). Those with a profound intellectual disability were less likely to show challenging behaviour over time (*B* = −1.275 [−2.004, −.545], *p =* .001).

Whether the child was a child in care, gender, English as language spoken at home, and neighbourhood deprivation were not associated with any challenging behaviour in the analysis models. Statistics for these variables can be seen in Table 3.

## 4 DISCUSSION

There was a high prevalence of challenging behaviour which did not vary significantly over the four annual time points assessed in this research. These are unique data and results suggest that challenging behaviour is relatively stable during school age, although stereotypy did slightly increase over time. The different time‐related patterns for sub‐types of challenging behaviour reinforce the need to examine challenging behaviour longitudinally by sub‐type.

Using a longitudinal design, with four time points of data, there was evidence that lower levels of adaptive skills were associated with increased likelihood of all types of challenging behaviour. Overall challenging behaviour increased with age, although this was likely related to increasing stereotyped behaviour and not other types of challenging behaviour. All subtypes of challenging behaviour were more likely when autism was present. Children with a profound intellectual disability were less likely to show self‐injurious behaviour over time and were also less likely to show overall challenging behaviour over time.

These findings are consistent with findings about correlates from previous cross‐sectional challenging behaviour research. For example, Holden and Gitlesen (2006) concluded that severity of intellectual disability was a risk factor for challenging behaviour in a total population study with adults. McClintock et al. (2003) found that autism, lower adaptive skills and communication skills were associated with challenging behaviour in an adult population. Mazurek et al. (2013) concluded that aggression is prevalent in children and adolescents with autism more so than in the general population. Lundqvist (2013) concluded that the presence of autism increased the risk of challenging behaviour. Nicholls et al. (2020) studied a special school population and reported that the presence of autism, lower level of adaptive skills and deprivation experienced by the family were associated with challenging behaviour in a cross‐sectional analysis.

The findings from the current study are also consistent with the limited longitudinal research. Chadwick et al. (2005) found there was little difference between the rate of aggression, destructive behaviour and self‐injury between the two assessment points, showing that challenging behaviour is stable over time. They found that this was stable from childhood through to early adolescence similar to the current study.

Furthermore, Davies and Oliver (2016) concluded that aggression, destruction and self‐injury in children was persistent over two time points 15–18 months apart. Although severity of intellectual disability was not significantly associated with challenging behaviour, they concluded that this was due to the small range and severe level of intellectual disability.

Rojahn et al. (2016) found that challenging behaviour persisted over time in younger children – consistent with the pattern found here of little change in prevalence of challenging behaviour over four annual time points. They also concluded that children who displayed more frequent self‐injurious behaviour at the later time point had showed more frequent stereotyped behaviour at the earlier time point showing this was influential. Given the increase in stereotyped behaviour in the current study, this would be worth considering how interventions could be identified for this group of children.

As far as we are aware, the current research over four annual time points is unique in relation to children with intellectual disabilities, but the study is limited to the population of one special school. Caution should be used when interpreting the data as the findings may not be generalisable to other school populations. However, a methodological strength is that this research could be easily replicated in other school populations; the methodology used is clear and replicable. A further caution in interpreting the data is that there was a limited range of fixed risk factors explored. This research used a naturalistic opportunity to gather relevant data, but the data collection exercise was not designed to consider all possible risk factors. Due to this, there were no data on medical or psychiatric comorbidities which may have had an impact on behaviours that challenge, and it would have been useful to look at these as other potential risk factors. There were also no data on the presence of life events such as moving house, or loss of family member or loved one that may have had an impact on behaviour. Further research is needed to examine other correlates and strengthen the evidence base. This study also did not account for any interventions that may have taken place over the period of the research.

This research adds to longitudinal evidence for risk correlates of challenging behaviour. The risk factors identified support the ongoing need for proactive interventions for those at high risk; especially those with autism, and those with poorer adaptive skills. For example, schools should probably prioritise interventions focused on supporting skill development including adaptive skills and communication as this could then reduce the risk of challenging behaviour. Teaching such skills is also crucial for children's development and should be embedded in the curriculum for children with intellectual disabilities in special school contexts.

## FUNDING INFORMATION

This research was completed as part of a PhD partially funded by Thrive Education Partnership.

## CONFLICT OF INTEREST

I am part of the senior leadership team in the school where data were collected. The other authors have no conflicts of interest to declare.

## Data Availability

The data that support the findings of this study are available from the corresponding author upon reasonable request.
